# Transposing musical skill: sonification of movement as concurrent augmented feedback enhances learning in a bimanual task

**DOI:** 10.1007/s00426-016-0775-0

**Published:** 2016-05-27

**Authors:** John Dyer, Paul Stapleton, Matthew Rodger

**Affiliations:** 1 School of Psychology, Queen’s University Belfast, David Keir Building, 18-30 Malone Road, Belfast, BT9 5BN UK; 2 Sonic Arts Research Centre, Queen’s University Belfast, Cloreen Park, Belfast, BT9 5HN UK

## Abstract

Concurrent feedback provided during acquisition can enhance performance of novel tasks. The ‘guidance hypothesis’ predicts that feedback provision leads to dependence and poor performance in its absence. However, appropriately structured feedback information provided through sound (‘sonification’) may not be subject to this effect. We test this directly using a rhythmic bimanual shape-tracing task in which participants learned to move at a 4:3 timing ratio. Sonification of movement and demonstration was compared to two other learning conditions: (1) Sonification of task demonstration alone and (2) completely silent practice (control). Sonification of movement emerged as the most effective form of practice, reaching significantly lower error scores than control. Sonification of solely the demonstration, which was expected to benefit participants by perceptually unifying task requirements, did not lead to better performance than control. Good performance was maintained by participants in the Sonification condition in an immediate retention test without feedback, indicating that the use of this feedback can overcome the guidance effect. On a 24-h retention test, performance had declined and was equal between groups. We argue that this and similar findings in the feedback literature are best explained by an ecological approach to motor skill learning which places available perceptual information at the highest level of importance.

**Electronic supplementary material:**

The online version of this article (doi:10.1007/s00426-016-0775-0) contains supplementary material, which is available to authorized users.

## Introduction

### Movement sonification and the guidance hypothesis in perceptual-motor learning

Concurrent augmented feedback is perceptual feedback about a movement which is presented live, alongside and during motor performance. It has been used successfully to enhance acquisition and learning in a wide range of motor tasks (Sigrist, Rauter, Riener, & Wolf, 2013a). However, learners typically become dependent on augmented information and performance declines when it is withdrawn (Park, Shea, & Wright, 2000; Schmidt & Wulf, 1997; Schmidt, 1991; Sigrist, Rauter, Riener, & Wolf, 2013b; Vander Linden, Cauraugh, & Greene, 1993). The high level of performance seen in the presence of concurrent feedback rarely persists into no-feedback retention tests, which constitute a truer test of learning (Salmoni, Schmidt, & Walter, 1984). The explanation for this is that learners come to rely too heavily on the augmented information provided by concurrent feedback, and ignore task-intrinsic sources of sensory feedback, an effect known as the ‘guidance hypothesis’ (Adams, 1971). Once augmented feedback is removed, the learner must rely on comparatively unfamiliar sources of intrinsic feedback (e.g. proprioception) and performance declines as a result of impaired performance-monitoring ability (Anderson, Magill, Sekiya, & Ryan, 2005). Intrinsic sources of sensory feedback may be unattended when augmented feedback is available for two possible reasons. The feedback display may simply distract attention from otherwise available intrinsic information, or it may provide performance information which is much easier to use than intrinsic sources.^1^

Emerging evidence suggests, however, that the guidance hypothesis is not a general principle of feedback as had previously been assumed (Danna et al., 2015; Mononen, Viitasalo, Konttinen, & Era, 2003; van Vugt & Tillmann, 2015; for a review, see Dyer, Stapleton and Rodger, 2015). Experiments using concurrent feedback in the auditory modality have shown that speed of acquisition can be enhanced using sound without impairing performance on subsequent no-feedback retention tests (Kennedy, Boyle, & Shea, 2013; Ronsse et al., 2011). Digitally transforming human movement data into sound (termed ‘sonification’ of movement) has long been practiced in the field of Sonic Arts as a method of musical expression (Hermann, Hunt, & Neuhoff, 2011; Medeiros & Wanderley, 2014). Recently Sonification of movement has emerged in the motor skill learning literature as a viable alternative to visual display for the presentation of concurrent augmented feedback, occasionally overcoming the limitations associated with feedback presented in the visual modality (Effenberg, 2005; Sigrist et al., 2013a).

For example: Mononen, Viitasalo, Konttinen, and Era (2003) sonified one-dimensional aiming error in rifle training by mapping positional error of the gun barrel to sonic pitch. Their participants, therefore, had access to an additional layer of performance-relevant information through sound and performance was improved as a result. Unlike concurrent feedback experiments in the visual modality, no decline in performance was observed following the removal of augmented feedback. The enhancement effect of feedback was maintained on no-feedback retention tests, even several days later.

Ronsse et al. (2011) tell a similar story and provide a rare example of visual and auditory concurrent augmented feedback contrasted on the same experimental task (90° out-of-phase bimanual flexion/extension). Concurrent visual feedback was provided in the form of a Lissajous figure (which draws a circle from perfect performance of a 90° phase relationship) and auditory feedback via Sonification of changes in wrist direction, which results in a ‘galloping rhythm’ when movements are performed accurately. They found that although visual feedback allowed learners to reach optimal performance more quickly than auditory feedback, this high level of performance was maintained only by the auditory group in no-feedback retention. A typical guidance effect was found following the removal of visual feedback, but not auditory feedback. Heitger et al. (2012) replicated the behavioural findings of Ronsse et al. using the same bimanual task.

These findings represent a slight challenge to traditional interpretations of the guidance effect, which assume that feedback presented 100 % of the time during acquisition will lead to decline when it is withdrawn because intrinsic proprioceptive feedback has been attentionally neglected (Anderson et al., 2005; Sigrist et al., 2013a). However, these results make a lot of sense from a broad ecological perspective. A possible explanation for the apparent advantage of sonification will be elaborated in the following sections.

### An ecological perspective on the guidance effect in bimanual tasks

If we consider motor control and learning to be a purely perception–action phenomenon (Fowler & Turvey, 1978; Gibson, 1969), the difference between visual concurrent feedback and sonification becomes more clear. The perceptual information about performance available to a learner during acquisition of a novel motor skill has broad implications for performance and retention. From an ecological perspective, attaining a skilful or accomplished level of performance in a given task is characterised by perceptual refinement (Michaels & Carello, 1981), wherein an individual gradually tunes into (and acts to produce) perceptual information within a range which specifies good motor performance. Concurrent feedback enhances motor performance by making such task-relevant perceptual information more salient or accessible (Wilson, Collins, & Bingham, 2005). The challenge for a learner is to learn how to use this information in the context of the task goals.

Bimanual coordination tasks are an ideal vehicle to probe these processes, as level of task difficulty is clearly defined in terms of either phase relationship (Kelso, Scholz, & Schoner, 1986) or polyrhythmic timing ratio (Summers, Rosenbaum, Burns, & Ford, 1993). In bimanual coordination tasks, the perceptual information associated with good performance (i.e. phase relationship or polyrhythmic ratio) is not clearly specified through intrinsic feedback alone, making these tasks extremely difficult to learn without concurrent feedback to make the information more available—typically via a visual Lissajous plot (Kovacs, Buchanan, & Shea, 2009; Kovacs & Shea, 2011; Wang, Kennedy, Boyle, & Shea, 2013). The effects of concurrent feedback on bimanual coordination tasks are, therefore, very strong (Kovacs, Buchanan, & Shea, 2010).

Motor learning in bimanual coordination tasks is clearly perceptually based^2^ (Franz, Zelaznik, Swinnen, & Walter, 2001; Mechsner, Kerzel, Knoblich, & Prinz, 2001; Wilson, Snapp-Childs, Coats, & Bingham, 2010). Bimanual coordination performance is so difficult to perceive intrinsically that learner attention is occupied entirely by controlling the feedback display; this is by far the most valuable information that the environment offers in the context of the task—and guidance effects are the norm (Kovacs et al., 2009; Kovacs & Shea, 2011). In this situation, the learner does not actually learn to produce the bimanual task; he/she learns how to manipulate the Lissajous display. This is demonstrated by Kovacs et al. (2010) who found that removing vision of the limbs allowed participants to very quickly learn to produce a 5:3 bimanual ratio—a feat previously thought to be impossible without extensive practice. Removing vision of the limbs may have helped because it streamlined/refined the perception–action loop to a single stream: perception of the dot’s movement and control over that action. As far as the learner was concerned, removing vision of the limbs relegated them to a plane of total non-existence, as the brain effectively adopted direct control over the movement of the dot (Swinnen & Wenderoth, 2004). It is very difficult to perceive useful information about bimanual coordination from the limbs themselves, and in fact any such information may actually conflict with the Lissajous information, as argued by Kovacs et al.

The guidance effect then comes as no surprise. In the case of visual feedback, the display *is* the task. This fact is not of great concern if one’s goal is to push the limits of perceptual control of action (Kovacs et al., 2010), but it is a real problem if the aim is to produce learning which transfers outside the lab. If the only way (or, the most effective way) for the learner to perceive their performance is through an augmented feedback display, then he/she will not be able to perform the task in its absence. In the next section, movement sonification will be examined from the same perspective.

### Noisy events, perceptual unification and sonification

Sonification is (or rather, can be) more than just another method for abstract display of symbolic movement data (Roddy & Furlong, 2014). There are distinct perceptual and phenomenological qualities of sound perception which may make it a more appropriate modality for meaningful concurrent feedback than a visual display (Dyer et al., 2015). These qualities can explain sonification’s potential immunity to the guidance effect.

Sound is intrinsically linked to movement (Leman, 2008; Repp, 1993; Sievers, Polansky, Casey, & Wheatley, 2013). In everyday life, sounds automatically become part of multimodal event perception (Gaver, 1993). Thanks to our extensive interactive experience with a noisy environment, we can perceive a surprising amount of action-relevant information from an auditory event (Giordano & McAdams, 2006; Houben, Kohlrausch, & Hermes, 2004; van Dinther & Patterson, 2006; Young, Rodger, & Craig, 2013). In the case of sounds produced by action, fMRI studies during passive listening have recorded neural activations similar to those observed during previous action performance (Kohler et al., 2002; Lahav, Saltzman, & Schlaug, 2007). Behavioural effects are especially strong for extensively practiced noisy actions, for example instrumental performance (Taylor & Witt, 2015). Additionally, specific actions can even be identified from their sonified velocity profile alone (Vinken et al., 2013). Summarised, sound and movement are ecologically coupled. Sound is inherently meaningful to the moving individual, and if it were employed as concurrent augmented feedback in a motor skill learning study, the link between participant movement and feedback could potentially be much tighter, and feedback less of an abstraction. In other words, sound as feedback is more coupled to fundamental task kinematics than a visual display. The use of sound can perhaps more explicitly include the body in the perception–action loop.

As shown by Ronsse et al. (2011) and Kennedy, Boyle and Shea (2013), auditory models/demonstrations of bimanual task performance along with sonification as feedback are effective for training complex coordination tasks. Making perceptual information about bimanual task performance more salient or perceivable leads to reduced variability in associated action, as shown by Wilson, Collins, and Bingham (2005). This seems to be a general perceptual effect which also applies to sound information and unimanual tasks. van Vugt and Tillmann (2015) found that accurate sonic feedback improved tapping accuracy in a learned motor task to a greater degree than jittered feedback. Interestingly, improved performance in the sonification group persisted into no-feedback retention and transfer tests. The temporal resolution of the auditory system is known to be much finer than that of the somatosensory system (Hirsh & Watson, 1996; Tinazzi et al., 2002), so one would expect more accurate temporal perception of any event paired with sound. Following an ecological approach to motor learning (Gibson, 1969), and assuming that perception never happens in isolation from action, it stands to reason that enhanced perceptual acuity for action’s consequences (i.e. feedback) will necessarily result in better control of action.

Ronsse et al. show that, although slightly slower, sonification is as effective for teaching a novel coordination pattern as the more commonly used Lissajous figure. Lissajous feedback works through perceptual unification, a transformation wherein a difficult bimanual task is consolidated and abstracted to create a new, more coherent and unitary percept (for the effect of perceptual unification on other bimanual tasks without Lissajous feedback, see Franz et al., 2001; Mechsner et al., 2001). Unification makes relevant perceptual information about the higher-order variable of relative phase/timing ratio more available, which allows effective and stable action production. We argue that a demonstration through sound functionally does the same thing; it consolidates a dual-task into a rhythm, which can be perceived and reproduced as a single action.

The potential advantage of sonification over Lissajous as *concurrent* feedback lies in the degree of abstraction, or transformation. As argued earlier, and presupposing good sound design,^3^ Sonification of bimanual coordination does not entail the same degree of transformation as does feedback displayed as a Lissajous figure, the Gestalt form of which differs substantially from the underlying kinematics of bimanual coordination. By contrast, sonification is layered on top of and can be used to emphasise relevant task kinematics. This can allow direct perception of phase relationship or timing ratio without subsuming the main motor task, as recommended by Wilson et al. (2010). Information about the higher-order relationship between the hands is present in task-intrinsic proprioceptive feedback; we should be able to use sound to train participants to perceive it directly—eliminating the guidance effect of concurrent feedback.

Our aim in this paper is twofold. First, we aim to further scientific understanding of the guidance effect of concurrent feedback, specifically how it relates to sonification. Second, we aim to separate the effects of perceptual unification from feedback to test whether unification of the task goals (through adding sound to the demonstration) is sufficient to enhance learning, or whether there is a distinct advantage of sonification as concurrent feedback. At this point, it is not yet clear whether the effects of sound on learning in Kennedy et al. (2013) are due to either perceptual unification through a sonic demonstration, or concurrent movement sonification. Performance in bimanual coordination is improved by perceptual unification alone (Franz & McCormick, 2010; Franz et al., 2001), and it will be important to establish this difference going forward. After all, one need not provide online Sonification of movement during practice at all if performance can be enhanced to the same degree using a pre-recorded, sonified demonstration.

To this end we have designed a novel bimanual shape-tracing apparatus to teach participants to produce a 4:3 rhythmic coordination pattern, a task previously shown to be difficult to learn (Summers et al., 1993).

We hypothesise that the use of sonification as auditory feedback will not lead to a guidance effect relative to no-sound control. Like Lissajous feedback, sonification represents a method to perceptually unify a bimanual task; however, it does not rely on a transformation and abstraction of the fundamental task kinematics. For this reason, we expect both enhanced performance of the sonification group during practice, and maintenance of this enhanced performance into retention-without-feedback.

We additionally hypothesise that performance in the condition in which the demonstration alone is sonified (hereafter referred to as the ‘sound-demo condition’) will benefit from the use of sound to perceptually unify the task demands, which will manifest as enhanced performance during practice and into retention relative to no-sound control.

We will also compare between the sound-demo alone and sonification as concurrent feedback. Both conditions perceptually unify the task demands, however, live sonification may confer a relative advantage in the acquisition stage by enhancing online temporal perception of performance. Improved perceptual acuity through sound should, in general, manifest as better performance (Fowler & Turvey, 1978), and we expect to see as much in this task, good performance in which is based at least partly on fine temporal control.

## Methods

### Participants

An opportunity sample of 45 right-handed participants [20 female; mean age = 24.3 years (SD = 5.9 years)] was recruited from a combination of undergraduate Psychology students, postgraduate researchers and staff at the university in which the experiment was conducted. Undergraduate students received course credit for their participation. Right-handedness was confirmed for all participants by administration of the Edinburgh Handedness Inventory (Oldfield, 1971). Handedness scores did not differ between experimental groups [*F*(2,42) = 0.335, *p* = 0.717].

Participants were questioned about their musical experience after completion of the study to avoid experimenter bias. Almost half (21 of 45 participants) reported some experience playing musical instruments, in most cases not currently. Eight participants in the Sonification condition reported musical experience, only one of whom was active. The other seven reported having ceased playing an average of 5.4 years ago. There were six musical participants in the Control condition, four active, the rest having ceased mean 5.5 years ago. The Sound-Demo condition contained seven musical participants, one active, with the rest having ceased mean 3.5 years ago.

Informed consent was obtained from all individual participants included in the study. All procedures performed in studies involving human participants were in accordance with the ethical standards of the institutional research committee and with the 1964 Helsinki declaration and its later amendments or comparable ethical standards.

### Materials and apparatus

#### Hardware

**Fig. 1:**
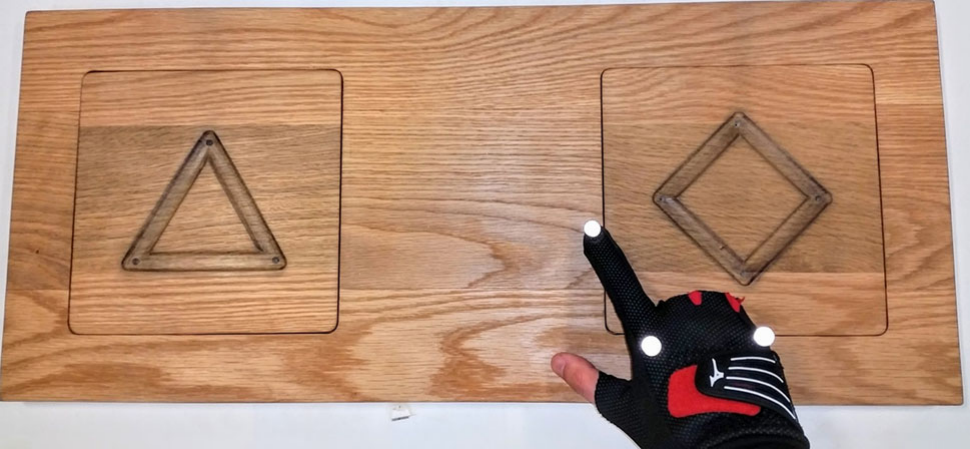
Participants traced the index finger of both hands around the shapes simultaneously in an *anticlockwise* direction, starting from the top corner

A bespoke wooden board (70 cm × 30 cm) was created for the purpose of this experiment (see Fig. 1). Two 20 cm × 20 cm slots were cut into the top side of the board, into which were inserted a pair of wooden slabs. On each of the slabs was carved a regular polygon (a diamond on one and a triangle on the other) of equal path length (34 cm). Shape grooves were rounded with 3 mm depth (at the centre) and 12.5 mm width. The board was placed on a desk at which participants were seated. Participant movement data were obtained using a Qualisys optical motion capture system capturing at 300 Hz, which was triggered using an Arduino controller. Participants wore a pair of modified golfing gloves with reflective markers attached, allowing the movement of the hands and tip of the index finger to be tracked in 3D space.

A 17-inch screen was used to display a demonstration animation corresponding to exemplary performance and a pair of Sennheiser headphones were worn by participants at all times. The experiment was administered by the experimenter using a desktop PC running Qualisys Track Manager (QTM).

#### Software

Data corresponding to participant movement in Cartesian space (*x*, *y* and *z*) were streamed in real-time from QTM to Max/MSP 6.0 via the OSC protocol. An exemplary demo animation and graphical display were programmed using *Processing.*

#### Sonification and terminal feedback

**Fig. 2:**
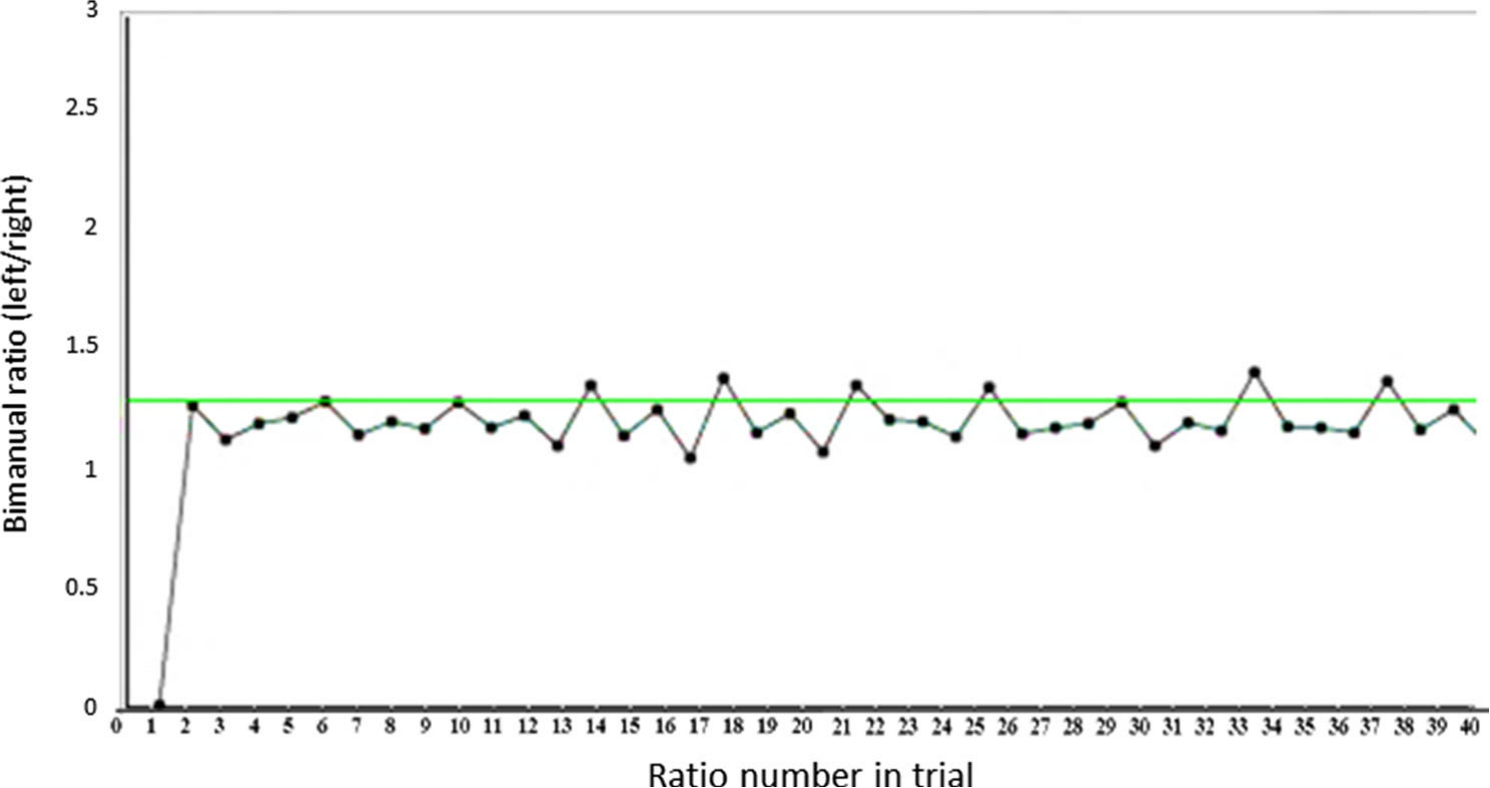
Intermanual ratio was continuously plotted on a graph which also showed the ideal 4:3 (1.33) ratio as the horizontal midline. The graph shown corresponds to relatively good performance (low error magnitude and variability). *Axes* labels were not visible to participants

In this experiment, participants engaged in a series of discrete practice trials, following which, post-trial (terminal) feedback was provided. A 3 × 3 cm (9 cm^2^ area) range was defined for each corner of the diamond and triangle shapes (i.e. a square, centered on each corner, boundaries extending 1.5 cm bi-directionally in the *x* and *y* planes), based on the position of the index finger marker (*x*, *y*) when a participant’s fingertip was positioned in the corners. A trigger was produced in Max/MSP by index finger arrival in any of these zones. An inter-trigger interval (time between corner arrivals) was thus calculated for the left and right hand. Each new right-hand interval was compared to the previous interval for the left hand to calculate a ratio (with the target right-to-left duration ratio of 3:4). These ratios were stored and displayed on a graph at the end of each practice trial as terminal feedback (see Fig. 2).

**Fig. 3:**
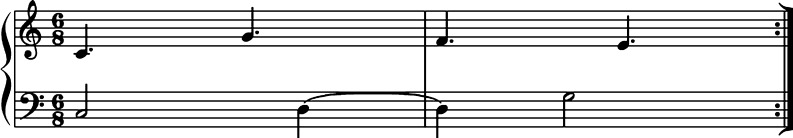
The left and right hand corner arrivals were sonified using synthesised tones not associated with any real-world instrument. The left (*bottom*) and right-hand (*top*) movements were unified into a single melody when the task was performed correctly

These same arrival triggers were used as the basis for concurrent sonification feedback. This model of sonification draws some inspiration from Ronsse et al. (2011), who sonified reversals in direction in a bimanual task; the endpoint of a movement trajectory was judged to be a salient perceptual event in both Ronsse et al. and the current experiment, and tightly-linked to the main goal of the task, i.e. timing. In the current experiment, each endpoint of a movement trajectory (i.e. arrival at a given shape corner) was represented by one of a set of notes in the key of C Major. Tones were generated in Max*/*MSP by combining a pure tone (with a given frequency corresponding to one of the notes in Fig. 3) with a predefined envelope function which modulated loudness over time. Following a trigger which initiated the tone, loudness decayed roughly exponentially, reaching silence after 350 ms.^4^ The notes for the left and right hand were taken from separate but adjoining octaves, as a close pitch relationship has been shown to be conducive to auditory “stream” formation and perceptual integration (Bregman & Campbell, 1971; Flowers, 2005). Thus, a short melody was played by correct performance of the task (see Fig. 3).

### Procedure

**Fig. 4:**
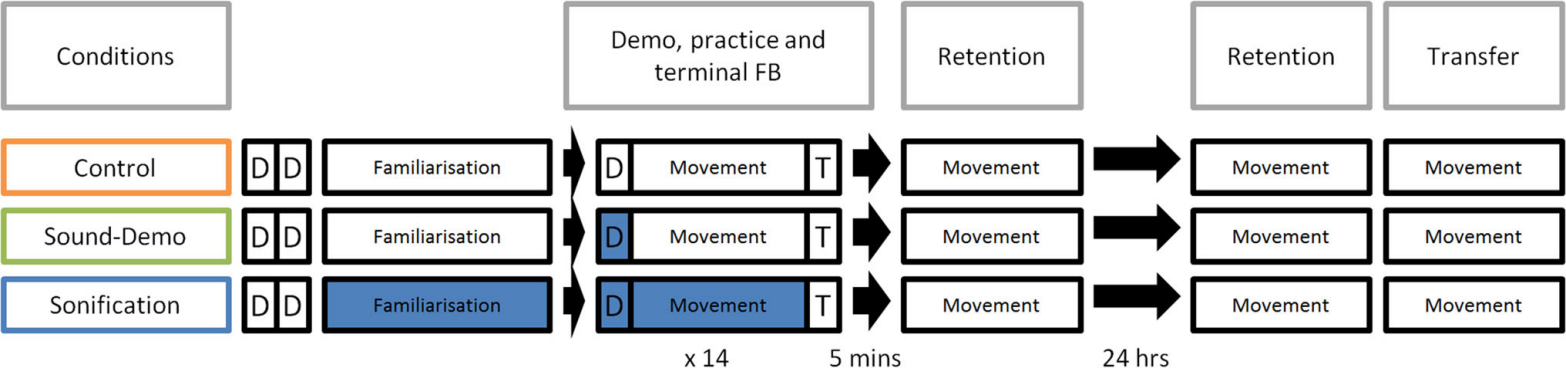
Experimental procedure. *Boxes marked* *D* represent a presentation of the demo animation. *Boxes marked* *T* represent terminal (*graph*) feedback. *Blue*/*shaded boxes* indicate the presence of sound at corner arrivals/sonification. All *unshaded* movement and demo sections occurring after familiarisation were paired with constant pink noise

Participants were pseudorandomly allocated to one of three conditions: Control, Sound-Demo and Sonification (*N* = 15 each). Each of these conditions entailed different availability of sound to guide performance. For a graphical visualisation of the entire experimental procedure, please refer to Fig. 4.

#### Familiarisation

The experiment began with a short task-familiarisation phase in which participants in all three conditions were shown a soundless visual demo animation of correct task performance. The demo showed two shapes on-screen (corresponding to the wooden shapes in front of the participant). Individual corner zones of the animated shapes lit up in sequence, demonstrating the spatio-temporal characteristics of the required 4:3 bimanual coordination ratio (Hove & Keller, 2010). One full cycle of the demo lasted 3 s (a left inter-trigger-interval of one second, right 750 ms). Three rotations were presented on each ‘play’ of the demo. Participants were played the demo twice during this familiarisation phase (comprising six rotations in total), then given approximately 15 s movement time, in which they attempted to reproduce the spatiotemporal characteristics of the movement seen in the demo. Participants in the sonification condition had their hand movements sonified during this time which served as familiarisation with the action-sound mapping; however, no participants had access to an audible demo at this point.

#### Practice

The practice phase consisted of 14 discrete trials for all participants. Each trial began with a play of the demo (9 s), followed by a movement phase (26 s), and concluded with presentation of terminal feedback (graph of bimanual ratios over time—Fig. 2).

The Control condition saw a purely visual demo and listened to constant pink noise during its presentation. During the movement phase for the Control condition, no sonification was provided—only constant pink noise was heard. Pink noise was used (at low volume) during the movement phase to mask any naturally occurring sounds from hand movement over the apparatus. Trials concluded with the graph presented as terminal feedback.

The Sound-Demo condition saw a visual-acoustic demo at commencement of each practice trial, in which corner arrivals were sonified using the tones shown in Fig. 3, without pink noise. During the movement phase, participants heard constant pink noise. Trials concluded with the graph.

The Sonification condition saw the same visual-acoustic demo as the Sound-Demo condition at commencement of each practice trial, without pink noise. During the movement phase, arrivals of the index fingers at corner zones were sonified using the procedure described earlier and the notes in Fig. 3. Perfect performance of the 4:3 ratio would produce the same melody heard in the demo. No pink noise was heard during movement. Trials concluded with the graph.

#### Retention

After 14 practice trials, all participants were given a five-minute break before undergoing a 26-s retention test without any augmented feedback (i.e. no graph and no sonification—where applicable). No demo was played prior to this trial. Participants in all three conditions heard pink noise during the movement phase. The retention test was repeated exactly on the following day.

#### Transfer

Last, a transfer test was administered to assess whether task learning would generalise to a differing degree based on the mode of learning. The application of learned motor skill to a different task context is generally taken as an indicator of robust learning (Soderstrom & Bjork, 2015). We tested transfer by switching the positions of the shapes to be traced. The task was essentially the same; 4:3 rhythmic coordination, only mirrored.

## Results

### Bimanual ratio of timing

Bimanual timing ratio was calculated continuously for each trial by comparing every right hand inter-trigger interval to the most recent interval for the left hand. This raw information was presented to participants as terminal feedback. For analysis, the difference between the values of these obtained ratios and the ideal (4:3) ratio was calculated, yielding a measure of absolute error over time. The mean of absolute ratio error served as a measure of performance for each trial, with a value of 0 indicating trial performance which perfectly matched the target ratio throughout.

### Average absolute bimanual ratio error in practice, retention and transfer trials across feedback groups

A mixed ANOVA on acquisition data (trials 1–14) with condition as a between-groups factor and trial as a repeated measures factor revealed a significant main effect of condition: *F*(2, 39) = 6.75, *p* = 0.003, *η* ^2^ = 0.137 and trial: *F*(5.098, 198.804) = 12.29, *p* < 0.001, *η* ^2^ = 0.120. No trial × group interaction was detected: *F*(10.200, 0.298) = 0.423, *p* = 0.936. Pairwise comparisons of inter-group score differences were performed at Trial 14 only to test whether there was a significant benefit of sonification by the end of practice. Alpha was set at 0.016 (Bonferroni correction for three comparisons). The Sonification condition performed the task with significantly lower error than the Control condition (*p* < 0.001, Cohen’s *d* = 1.344), but not the Sound-Demo condition (*p* = 0.031, Cohen’s *d* = 0.839). No difference in scores was evident between the Sound-Demo and Control conditions (*p* = 0.757). Participants who learned with sonification were evidently better at the task on the final practice trial than their counterparts in the Control condition.

To identify differences in rates of learning, we performed a linear regression with Trial as predictor on the data from trials 1-14 for each of the three conditions. We found significant models in all three conditions. For the Sonification condition: *F*(1,207) = 42.20, *p* < 0.001, the Control condition: *F*(1,206) = 21.672, *p* < 0.001, and the Sound-Demo condition: *F*(1,205) = 19.88, *p* < 0.001. Trial significantly predicted task performance in the Sonification condition *β* = −0.41, *t*(14) = −6.50, *p* < 0.001, the Control condition *β* = −0.31, *t*(14) = −4.67, *p* < 0.001 and the Sound-Demo condition *β* = −0.30, *t*(14) = −4.46, *p* < 0.001. The standardised *β*-coefficients presented here indicate very similar rates of learning in the Control and Sound Demo conditions (−0.31 and −0.30, respectively) with slightly faster learning in the Sonification condition (−0.41).

One of our primary interests in the current experiment is in the presence (or absence) of a guidance effect after the removal of sonified augmented feedback. We need to be able to tell whether the improved performance in the Sonification condition was dependent on the presence of feedback, and whether it deteriorated after it was removed. To this end, we test for statistical noninferiority of Sonification group error scores in the 5-min retention test relative to Trial 14. This procedure is described in full by Walker and Nowacki (2011). In brief, if the 90 % confidence interval (CI) of the difference scores (between trial 14 and 5-min retention) falls within a pre-set noninferiority interval, then noninferiority of retention performance can be inferred at the 0.05 level. We set our noninferiority interval at 0.087, given that this is 0.5* the difference in mean scores between Sonification and Control conditions at trial 14. If the upper CI of the 5-min retention minus trial 14 difference scores falls below this value, then we can say that performance did not deteriorate (positive values indicate performance worsening in this arrangement). This is a common procedure for noninferiority testing in clinical drug trials in which noninferiority of a new drug (relative to an old drug) is inferred based on whether the 90 % CI of difference scores between a new drug and the old falls within an interval set by 0.5* the difference between the efficacy of the old drug and placebo (Walker & Nowacki, 2011, p. 194). The mean of the difference scores between Trial 14 and 5-min retention was 0.021, with a 90 % CI of [−0.041, 0.062], which means that performance was not inferior after sonification was removed. We are also able to provide a *p* value for the noninferiority test (as recommended by Walter and Nowacki) by performing a one-sided, one-sample *t* test on difference scores relative to the equivalence interval, 0.087: *t*(14) = −2.841, *p* = 0.013.

**Fig. 5:**
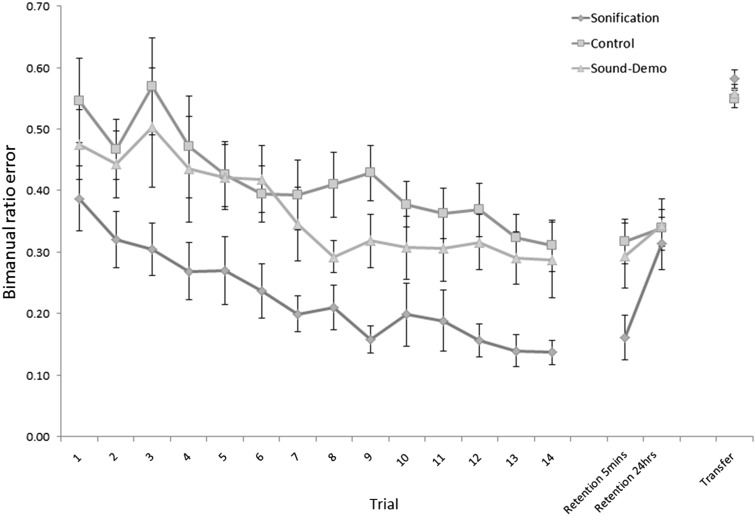
Rates of average absolute ratio error for the three feedback groups during practice, retention and transfer (Learning curves). A score of 0 represents perfect performance. Feedback was provided on trials 1–14. *Error bars* are standard error

On the second retention test, it is clear from Fig. 5 that the advantage of sonification had evaporated and performance had declined. Testing for group differences at this point revealed no main effect of condition *F*(2,42) = 4.15, *p* = 0.663, *η* ^2^ = 0.020, indicating that between-group performance had equalised at this point. Performance was similar on the transfer test, where no main effect of condition was present *F*(2,42) = 1.29, *p* = 0.287, *η* ^2^ = 0.054.

## Discussion

### Benefits of sonification for motor control in acquisition

By the end of acquisition, participants in the Sonification condition showed improved performance relative to Control (*p* < 0.001, Cohen’s *d* = 1.344), which indicates that concurrent sonic feedback was beneficial for acquisition (see Fig. 5). In this experiment, sound was used for two task-relevant purposes: one, to allow participants to directly perceive the higher-order variable which constituted the main goal of the task: bimanual timing ratio. This was accomplished by attaching tones to corner activations in the demo (and practice for the Sonification condition), creating a global melodic pattern (Franz & McCormick, 2010). Two, to more precisely specify (temporally speaking) the micro-level structure of the pattern i.e. the required timing of individual corner arrivals (and produced timing, in the case of Sonification). It has been shown that the temporal-perceptual resolution of proprioception is much lower than that of audition (Hirsh & Watson, 1996; Tinazzi et al., 2002), and we hoped that this could be augmented by exploiting sound to more clearly specify the temporal position of each corner-arrival. The performance data from the Sonification condition then conform to our hypotheses. Sonified participants had access to a both a unified percept of the required movement pattern and precise temporal specification of their performance, an arrangement which facilitated very fine-grained performance-monitoring and demo comparison. As can be seen in Fig. 5, the Sonification group showed improved performance relative to both other groups throughout the acquisition phase.

We also observed differences in rate of learning between experimental groups. Although the differences are small, *β*-coefficients from our regression analysis of performance data from trials 1–14 indicate that learning was indeed faster with sonification (−0.41) than without (−0.31), or with a sonic demonstration (−0.30). The lack of a stronger difference here may be due to a limitation of our experimental design, which does not include a true pre-test under identical experimental conditions across groups (see Fig. 4). Instead, the first trial for the Sonification condition included the presence of sound feedback, and the demo was immediately sonified in both the Sonification and Sound-Demo conditions. It is, therefore, inappropriate to treat the first trial as a pre-test or baseline measure of performance. Although performance on the first trial was extremely variable between participants, it is possible that an immediate first-trial advantage for Sonification was in play. This could have caused the learning curves to appear slightly more parallel and similarly shaped than they would have been had we included a true pre-test prior to trial 1.

Given the high informational value of sound in this context with regard to demonstration, the finding that there was no corresponding advantage evident in the Sound-Demo condition relative to Control by the end of acquisition was unexpected. Kennedy et al. (2013) found that practice with an auditory model led to lower error and variability than with a purely visual model, and we had to some extent expected the same, despite the confounder of concurrent auditory feedback in Kennedy et al. Instead, we found highly similar performance in the Sound-Demo condition to Control at trial 14 (*p* = 0.757), and similar rates of performance improvement from trial 1–14 (*β*-coefficients = −0.31 and −0.30 for Control and Sound-Demo conditions respectively).

The factor which differentiates Sonification then, is the availability of concurrent auditory information. Participants in the Sonification group completed fourteen 26-s-long trials of a novel, semi-musical movement task, which seems to have been enough practice to learn the mapping between action and sound. A merging of perception and action occurs in musical instrument training (Drost, Rieger, Brass, Gunter, & Prinz, 2005), such that actions are perceived in terms of their musical outcomes—and we maintain that a similar merging occurred here, despite the comparatively brief timescale. The movements of the motor task became causally associated with the co-occurrence of musical tones; this is a simple, tightly deterministic mapping not unlike that of a traditional musical instrument (for an explanation of how determinism in musical mapping affects comprehensibility, see Chadabe, 2002). In summary, the working mapping enabled participants to use auditory information to tell them about their motor performance.

This may explain why the Sonification condition showed an advantage in performance relative to Control when the Sound-Demo condition did not. The relatively low temporal acuity of proprioception as a feedback modality may have been a limiting factor for performance in the Sound-Demo condition, whereas proprioceptive feedback was augmented with sound in the Sonification condition. As predicted by a perception–action approach to motor control (Fowler & Turvey, 1978; Gibson, 1969), enhanced perception of action’s consequences leads to improved control of action.

We also expected to find a specific benefit of Sonification relative to the Sound-Demo by the end of practice (on trial 14). Although the difference between groups at this point was in the expected direction (ratio error of 0.14 and 0.29 in sonification and Sound-Demo, respectively), a post hoc *t* test did not quite reach statistical significance (*p* = 0.031, *α* = 0.016). This finding was unexpected but can perhaps be attributed to relatively high performance variability in the Sound-Demo condition at this time (SD = 0.24, compared to 0.08 in the Sonification condition), making statistically significant mean differences between the Sound-Demo condition and others more difficult to detect.

### The ‘guidance effect’ in early retention

A very similar pattern of results can be observed in the first no-feedback retention test as appeared on the final practice trial, when feedback had been available. Good performance by the Sonification group was shown to carry over into retention. Participants were able to overcome the guidance effect of concurrent feedback and maintain good performance without sonification. This result is in accordance with Ronsse et al. (2011) and Heitger et al. (2012), who also found no evidence of a guidance effect upon removal of auditory feedback in a bimanual task.

This finding suggests that participants had been trained to more accurately perceive the higher-order variable of timing ratio from their own intrinsic feedback, as we had expected. The movements of the task became associated with the production of a melody which specified the required timing ratio, essentially making retention a mute musical recital. It has been shown that musicians experience sounds associated with practiced musical actions when performing the actions in isolation (Lotze, Scheler, Tan, Braun, & Birbaumer, 2003), and that this audio-motor coupling can be induced in amateurs with relatively little practice (Lahav et al., 2007). This lines up well with post-experiment reports from participants in the Sonification condition, almost all of whom stated that they imagined playing the melody during the first retention test. van Vugt and Tillmann (2015) found that sonification of finger tapping resulted in improved timing accuracy and lower tapping variability (a result predicted by the perception–action approach to motor control invoked earlier), however, the benefit persisted even after the removal of sound. This implies that learned associations with sound may allow such experienced individuals to more accurately perceive temporal information in proprioceptive feedback, overcoming its intrinsic limitations. Thus, we maintain that a coalition of benefits associated with sonification were in operation in the current experiment to produce this result.

This study then adds to the growing literature on sonification and its apparent immunity to the guidance effect (Heitger et al., 2012; Mononen et al., 2003; Ronsse et al., 2011; Sigrist et al., 2013a; van Vugt & Tillmann, 2015).

### Action-sound mapping

The successful implementation of sonification as concurrent augmented feedback here is worth discussion in light of some other, more inconsistent findings. Despite the fact that this finding lines up with some other recent results from sonification experiments, (Heitger et al., 2012; Mononen et al., 2003; Ronsse et al., 2011; van Vugt & Tillmann, 2015), it may still be premature to say outright that sonification per se as concurrent feedback is immune to the guidance effect. To expand, one cannot always assume that substituting graphical visual feedback for sound will necessarily enable learners to perceive and use this information to an equal degree. The mapping between movement and sound must be carefully considered, especially since there is a crippling lack of overarching guidelines for mapping design. Sigrist et al. (2013b) for example, found no benefit of presenting several dimensions of rowing error through sonification. The authors assumed that since the sensory information was available (as variation in pitch, volume and stereo balance), participants would pick it up and be able to use it. This approach was not effective for motor control and learning. Granted, we know that listeners can perceive and distinguish between several streams of sonic information, given that they are mutually distinctive (Fitch & Kramer, 1994; Flowers, 2005). However in a motor learning experiment like Sigrist et al. (2013b), the challenge of perceiving how the information present in each of these streams covaries with motor performance could prove difficult in and of itself. Instead, a more effective approach—as far as comprehension is concerned—may be to aim for mappings that preserve the structure of intrinsic perceptual information (for a good example, see Stienstra, Overbeeke, & Wensveen, 2011). Sonification of movement (and indeed, concurrent augmented feedback generally) may be at its most effective when it is untransformed, i.e. structurally redundant with respect to the intrinsic perceptual information which needs to be controlled to perform the task competently in the absence of feedback. This may even be the factor which allows some forms of feedback to overcome the guidance effect (see Ronsse et al., 2011 for a comparison between transformed (Lissajous) and untransformed (sonification) augmented feedback).

Reports of the success or failure of sonification as feedback (including the current experiment) should be interpreted cautiously, and with awareness of these broader issues. For a wider discussion of the sonification mapping issue in the context of motor skill learning, see Dyer et al. (2015).

### Long-term retention and exploiting the musicality of movement

At the 24-h retention test, we observed no benefit of Sonification relative to Control or the Sound-Demo conditions. ANOVA revealed no significant effect of condition (*p* = 0.663) at this point, as performance in the Sonification group roughly equalled that of the two others. Reports from sonified participants at the time of this test indicated that most could no longer remember what the melody was supposed to sound like, and were keenly aware that their performance had declined from the previous day, despite receiving no feedback of any kind. It is thus, unsurprising that the same pattern of results is observed in the transfer test, which was conducted immediately following the 24-h retention. Participants had lost the ability to perform the base task, therefore, they were unable to apply their skill in a novel scenario.

This suggests that this 4:3 bimanual coordination pattern had effectively become a musical task. The ability of music to guide movement in a way which is aligned with more abstract task goals represents the fundamental (and currently underexploited) potential of sonification to train a wide range of otherwise non-musical skills (for a sonification prototype based on this line of thinking, see Kleiman-Weiner & Berger, 2006). From a motor-performance perspective, accomplished musical instrument performance represents one of the most impressively complex and temporally precise ways in which the human motor system can be deployed. This deployment is of course in service of a higher-order goal, the production of music; an accomplished performer is generally less concerned with the minutiae of motor control at the muscular level than the creation and maintenance of an overall Gestalt in the form of music. This is evidenced in the observation that the types of errors made by more advance-skilled musicians are those which are more likely to preserve the harmonic and temporal integrity of the musical whole (Drake & Palmer, 2000). Furthermore, we can be certain from the perceptual-motor literature discussed here that the precision of motor output evident in musical performance is afforded precisely because of the audio-motor link inherent in music. The recruitment of auditory perception in concert with a process of learning which enables an understanding of how one’s movement can alter sound, results in control of motor output which is unrivalled in most other domains of activity. The present experiment shows that potential exists for the exploitation of music in motor skill learning (through sonification), which in theory could be applied to many other skills that require precise control of movement, e.g. sport, or re-learning of basic skills in motor rehabilitation. If we can emphasise the latent musicality in skilled action, movement sonification could see broad applicability.

Further research should focus on ways to extend sonification’s guidance-effect immunity in time; we could speculate for example that refreshing a learner’s memory as to the exemplary sound profile might enable early retention-level performance to re-emerge, as perception of the sonic outcome of musical motor performance entails holistic perception of the movement event which precipitated it (Gaver, 1993; Lahav, Katz, Chess, & Saltzman, 2013; Lahav et al., 2007). Performance could thereby be enhanced without actually ever needing to re-expose participants to concurrent feedback.

Traditional musical instruments are entirely deterministic; the causal chain linking the movement of the performer with the sonic output of the instrument is entirely mechanical, and the mapping is therefore learnable with practice (Chadabe, 2002). By contrast, digitally mediated sonification of movement is not bound by these same limitations. There is therefore a very real risk of designing mappings which are inappropriate or ineffective. Future attempts to sonify movement for the purpose of performance enhancement should constrain the sound-design process to mappings which can provide the finer-grained information about movement that the learner might require to better control their action, and iterative pilot-testing of prototypes is essential.

## Conclusion

The main finding in the reported experiment concerns the guidance effect of augmented feedback as it applies to sonification. We have explained and shown that, under the right conditions, concurrent sonification can overcome the assumed dependency on feedback. We argue that this was possible by treating the task as a musical one, which allowed our participants to display some of the fine temporal and higher-level Gestalt control of movement commonly seen in musical instrument performance. Similarly to how accomplished piano players can produce reasonably accurate performances of well-known pieces without sound, our participants were able to perform the task in short-term retention. It is also interesting to note that the benefit of using sound for learning here was restricted to concurrent sonification; provision of a sonified demo alone did not improve performance relative to control.

## Electronic supplementary material

Below is the link to the electronic supplementary material.

Supplementary material 1 (WMV 12290 kb)

## Footnotes

1 The latter is likely the case for certain kinds of transformed visual feedback, which come to stand in for very difficult-to-use intrinsic sources (e.g. Kennedy, Wang, Panzer, & Shea, 2016; Mechsner et al., 2001).

2 This is likely also the case for motor skill learning in general. Bimanual coordination is not a special kind of learning except that the effects of manipulation of perceptual information are much more profound than in most other tasks.

3 Sound design is often given only cursory attention in perceptual-motor learning studies dealing with sonification. We will present a case for its importance in the discussion.

4 A video showing performance of the task and associated sonified feedback is available in the online supplementary materials for this paper.
